# A systematic review of types of healthy eating interventions in preschools

**DOI:** 10.1186/1475-2891-13-56

**Published:** 2014-06-06

**Authors:** Mette V Mikkelsen, Sofie Husby, Laurits R Skov, Federico JA Perez-Cueto

**Affiliations:** 1 Development of Planning and Development, Research group for Meal science and Public Health Nutrition – MENU, University of Aalborg, A.C. Meyers Vaenge 15, Copenhagen SV 2450, Denmark

**Keywords:** Preschool, Kindergarten, Healthy eating, Intervention, Obesity, Vegetable consumption

## Abstract

**Background:**

With the worldwide levels of obesity new venues for promotion of healthy eating habits are necessary. Considering children’s eating habits are founded during their preschool years early educational establishments are a promising place for making health promoting interventions.

**Methods:**

This systematic review evaluates different types of healthy eating interventions attempting to prevent obesity among 3 to 6 year-olds in preschools, kindergartens and day care facilities. Studies that included single interventions, educational interventions and/or multicomponent interventions were eligible for review. Included studies also had to have conducted both baseline and follow-up measurements.

A systematic search of the databases Scopus, Web of Science, CINAHL and PubMed was conducted to identify articles that met the inclusion criteria. The bibliographies of identified articles were also searched for relevant articles.

**Results:**

The review identified 4186 articles, of which 26 studies met the inclusion criteria. Fifteen of the interventions took place in preschools, 10 in kindergartens and 1 in another facility where children were cared for by individuals other than their parents. Seventeen of the 26 included studies were located in North America, 1 in South America, 5 in Asia, and 3 in a European context.

Healthy eating interventions in day care facilities increased fruit and vegetable consumption and nutrition related knowledge among the target groups. Only 2 studies reported a significant decrease in body mass index.

**Conclusions:**

This review highlights the scarcity of properly designed healthy eating interventions using clear indicators and verifiable outcomes. The potential of preschools as a potential setting for influencing children’s food choice at an early age should be more widely recognised and utilised.

## Introduction

The worldwide prevalence of overweight and obesity among preschool children has increased from 4.2% (95% CI: 3.2%, 5.2%) in 1990 to 6.7% (95% CI: 5.6% – 7.7%) in 2010 and is expected to increase even further to 9.1% (95% CI: 7.3 – 10.9) in 2020 [1]. This increase is disturbing due to the accompanying social, psychological and health effects and the link to subsequent morbidity and mortality in adulthood [2,3].

Considering the consequences of overweight and obesity on both a personal and societal level, healthier eating habits among children should be promoted as one of the actions to prevent overweight and obesity in future generations. The most common place for health promotion among children has previously been in the school setting mostly with children aged 6 to 12 years-old. But, there are promising findings in interventions targeting infants and 5-year-olds, although there is an underrepresentation of interventions and research within this age group [4]. Most of these interventions have been taking place in early education establishments for 3–6 year-olds like preschools in the U.S. or kindergartens as they are called outside the U.S. as well as daycare facilities, where children are nursed by a childcare giver in a private home. In this setting children consume a large number of their meals and may consume up to 70% of their daily nutrient intake [5]. These captive settings present a venue for intervention because institutional catering may be designed in such a way that nutritional guidelines are followed, resulting in an adequate food intake [6] and improved food choices later in life [7]. The objectives of the early educational establishments are often to teach and develop the child’s opportunities and skills that will prepare them for a better future [8] and many of the previous interventions have either focused on developing food preferences among children often by exposure or with nutritional educational interventions or with a combination of these two approaches. Previous reviews have included intervention studies that evaluated the outcomes of dietary educational interventions versus control on changes in BMI, prevalence of obesity, rate of weight gain and other outcomes like reduction in body fat, but as stated previously this did not yield a sufficient number of studies to provide recommendations for practice [4,9]. The Toybox study [10] has published a number of reviews about several aspects of health promotion efforts for pre-schoolers including the assessments tools of energy-related behaviours used in European obesity prevention strategies [11], the effective behavioural models and behaviour change strategies underpinning preschool and school-based prevention interventions aimed at 4-6-year-olds [12]. They also published a narrative review of psychological and educational strategies applied to young children’s eating behaviour in order to reduce the risk of obesity and found that there was potential for exposure and rewards studies to improve children’s eating habits [13]. None of the recent published studies have included both interventions that include both exposure or meal modification and educational interventions and multicomponent interventions that combine both approaches. With the exception of [13] all the previous reviews include physical activity and although this is an important factor in obesity prevention, many interventions do only focus on nutritional education and is as such excluded from previous reviews.

The objective of this article is to review published literature on healthy eating interventions in day care facilities and analyse the effectiveness of different strategies in relation to their influence on children’s food choice at an early age. Based on findings, this article also provides recommendations for future interventions.

## Methods

A systematic search for literature using four databases (PubMed, Scopus, Web of Science and CINAHL) was carried out. The search strategy was based on a careful selection of keywords and clear, pre-established criteria for inclusion of studies.

### Inclusion criteria

Included studies were intervention studies with the objective of treating or preventing the occurrence of obesity by influencing preschool children’s eating habits. As a prerequisite for inclusion, the healthy eating interventions had to take place in institutions and had to have taken both baseline and follow-up measurements. Although it is acknowledged that physical activity interventions are important and should not be disregarded, this study focuses solely on healthy eating interventions. Only studies targeting children aged 3 to 6 years were included as it is this age group that predominantly attends early education facilities. Since early education and school systems vary from country to country, it was decided to include all interventions in day care facilities if the mean age was between 3 to 6 years old. Children in included studies also had to be healthy at initial baseline measurement, although obese children were included in order to recognize the already existing prevalence of overweight and obesity in children and the necessity to acknowledge treatment of this particular target group. Interventions that focused on diet, nutrition, food, eating or meals in day care facilities were included. Due to the importance of environmental factors in children’s acquirement of healthy eating habits, interventions including kitchen employees and childcare givers in day care facilities were also included. As the review concerns itself with the effectiveness of different interventional strategies, the types of interventions were categorized into single component interventions, educational components, and multicomponent interventions that aiming to promote healthy eating habits and counteract obesogenic actions in children attending day care facilities.

The review included studies measuring biological, anthropometric and attitudinal outcomes: body mass index, z-scores for height and weight, waist to height measurements, serum cholesterol levels, skin-fold measurements or prevalence of overweight and obesity in the sample population, as well as food consumption patterns, knowledge and attitude towards foods and liking and willingness to try new food.

### Exclusion criteria

Research into weight loss of obese children and any interventions involving children with special needs or who were chronically sick and required on-going counselling, such as patients with diabetes or heart disease, were excluded from the review. Studies taking place in nursery, primary or elementary schools were also excluded when the mean age was either younger than 3 years or older than 6 years old. Interventions targeting parents of preschool children and descriptive articles about pre-schoolers behaviour, knowledge and consumption were also excluded. Lastly, studies including a physical activity component were excluded unless the dietary component was clearly separated from the physical activity intervention during implementation and analysis.

#### Conducting the search

Literature for the review was obtained using a systematic search conducted during spring 2014 with relevant literature published up to and including the search period. A meta-analysis was intended, however due to a lack of sufficient data, a meta-analytical comparison was difficult to deploy.

### Databases

The databases Scopus, Web of Science, CINAHL and PubMed databases were used for the literature search. The search was restricted to articles written in English, German, Norwegian, Swedish, and Danish as these were the language capabilities present in the reviewing group. The filter for research involving humans only was activated and the search was conducted to obtain articles published between 1980 and 2014.

The search strategy was created using relevant terms describing settings, possible inputs in an intervention and possible outputs of an intervention. The search terms were refined a number of times in order to optimize the selection of articles, without compromising with the sensitivity of the search in order to take into account the vast number of articles published on the topic of children and obesity. The keywords can be found in Table 1.

**Table 1:** Keywords for literature search

**Setting**	**Behaviour**	**Outcome**
**Kindergarten, preschool, day care facilities, nursery**	cooking ability, skill, or competence, food and nutrition literacy, curriculum or syllabus, teaching, taste development, food and meal policies, legislation or regulations or farm to fork or plate, garden farm or visits; food and taste education	BMI, body mass index, diabetes, skinfold, weight and height, and intervention, food neophobia and neophilia, food and meal preferences including liking, willingness to try, knowledge, food consumption or intake

#### Data management

**Figure 1:**
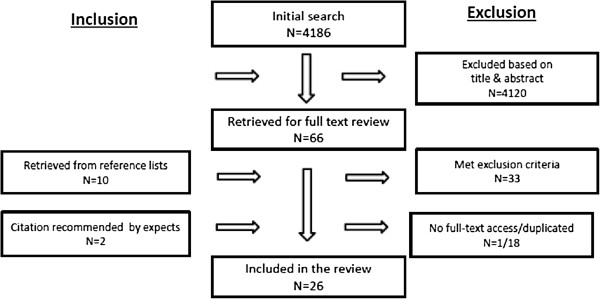
Flowchart of the study selection process.

The search hits were downloaded and saved in the databases. A total of 4186 papers were identified and screened on the basis of titles and abstracts by the first author, who has experience within a preschool venue, leaving 66 papers for further enquiries. Reference lists from the systematic review were scanned in order to identify interventions in kindergartens and preschools that the previous search had been unable to detect. Altogether, 10 papers were identified. After removing repeated studies and articles, 47 full text papers were retrieved through the library service at University of Aalborg, campus Copenhagen.The 47 remaining papers were read independently by three reviewers in order to verify that they met the inclusion criteria. 33 papers were excluded as a consequence primarily because they did not publish results, solely was targeted parents or were descriptive in nature. The reviewing process resulted in 26 papers left for analysis. Figure 1 contains an overview of the search process.

#### Data collection and analysis

##### Selection of studies

Articles identified in the literature search were read by the first author and divided between three reviewers for further evaluation and was debated in meetings with all three reviewers present.

For each of the located interventions, the following was extracted: aim of the study, setting where 3–6 year-olds were cared for by others than their parents, study design, characteristics of the target group, sampling methods, sample size, ethnicity, and theoretical background. Furthermore; duration, content and delivery mechanism of the intervention was extracted, as well as information about the control group, random allocation to control or treatment and whether there was information missing from the article.

#### Quality assessment

The quality of the identified studies was assessed using a rating scheme from * (weak) to **** (very strong). The studies were rated according to the level of information available, study design, risk of bias, study population and study duration. The quality rating scheme was adapted from the Cochrane guidelines on quality assessment [14]. Table 2 illustrates definition and explanation of the research design rating scheme. Each included study was rated independently among the three first authors (MVM, SH & LRS) with strong inter-rater reliability and disputes over assessment were settled through discussion.

**Table 2:** Quality rating scheme

**Rating**	**Definition**	**Study description**	**Design & methods**
*	Weak	Many details missing (three or more of the following: setting, intervention design, duration, intensity, population or statistical analysis) irrelevant design or methods	Methodological flaws (in statistical methods used or design of intervention) or the intervention was in a non-natural environment i.e. food laboratory
**	Moderate	One or two details missing	Small sample size (<50) or short duration (<one week)
***	Strong	One or two details missing	Larger sample size or longer duration
****	Very Strong	Clearly presented with all details provided	Larger sample size or longer duration and at least one of the following criteria: population randomly allocated or matched for intervention or control, generalizable results, or validated dietary assessment

Adapted from Seymour et al. [15]. Table 2 showing the standars for quality rating of the studies.

## Results

The 26 studies that the literature search resulted in were divided into 8 single intervention studies, 11 educational interventions and 7 multicomponent studies. The single intervention studies involved the modification of a single factor in the environment in order to promote fruit or vegetable intake and preferences in children. Educational interventions were carried out in the kindergartens, either by teachers that had undergone a teaching program or by nutritional educators provided by the research program and aiming to increase children’s knowledge of healthy eating. Multicomponent interventions included more than one strategy to influence eating behaviour.

Table 3 shows the characteristics of the studies.

**Table 3:** Characteristics of studies

**Study**	**Aim of study**	**Setting**	**Age (years)**	**Ethnicity**	**Detailed description of the intervention**	**Theoretical foundation**
**Single intervention**						
**Bannon et al.;**[16]	The purpose of the study was to develop and test a commercial for apples on kindergarten children’s snack choice.	Kindergarten in Elementary school (USA)	5^1^	92% Caucasian	The children were shown 60 s videos with either 1 positive gain-framed video; 1 negative loss-framed video; 1 control video prior to apple/animal crackers eating.	Not stated
				4% African- American 4% Hispanic		
**Birch et al.;**[17]	The influence of peer models’ food selections and eating behaviours on preschoolers’ food preferences was investigated.	Preschool (USA)	3,1^1^	87% Caucasians.	A target child who preferred vegetable A to B was seated with 3 or 4 peers with opposite preference patterns. 17 situations were arranged. Children were served their preferred and non-preferred vegetable pairs at lunch and asked to choose 1. On day 1 the target child chose first, while on days 2, 3, and 4 peers chose first.	Not stated
				8% African American		
				5% Asians, Middleclass families		
**O’Connell et al.;**[18]	The trial tested the hypotheses that children who are served unfamiliar vegetables repeatedly in the preschool lunch setting will increase consumption of them, and that consumption will be influenced by peer eating behaviours and parental feeding behaviours.	Preschool (USA)	4-5^2^	Caucasians: 69%	Serving of unfamiliar vegetables repeatedly in a preschool lunch setting 10 times during a 6 week period. Influence of between child variability and thus peer influence.	Not stated
				Asian 8%		
				African-American: 5%		
				Hispanic: 6%.		
				Other: 12%		
				From highly educated households.		
**Harnack et al.;**[19]	To evaluate the effects on serving vegetables first or together with the meal on fruit, vegetables and energy intake among preschoolers.	Preschool (USA)	Missing	Not stated	Meal service strategy: serving vegetables first, compared with serving all food items at the same time compared with control (no change). Every strategy was implemented in two weeks.	Not stated
**Hendy;**[20]	The purpose of the study was to examine the effectiveness of trained peer models to increase food acceptance of preschool children and the test whether the same gender would be the most effective.	Preschool (USA)	3-6^2^	90% Caucasians	Three novel foods presented during the preschool meal. 16 children were trained by their teachers to serve as peer models and given toy reinforcement.	Social cognitive theory.
				8% African American		
				2% Hispanic		
**Leahy et al.;**[21]	To test the effect of reducing the energy density of an entrée on children’s ad libitum intake.	Preschools, (USA)	3,9^1^	Caucasians: 69%	Children were served two version of a macaroni and cheese dish with the same palatability; one was energy dense and the other calorie-reduced. Each version was served 3 times.	Not stated
				Asian: 27%		
				African-American: 4%		
				90% of the mothers and 85% of fathers reported having at least a 4-year university degree		
**Noradilah;**[22]	The objective of this study was to determine the effects of multiple exposures to the acceptance of a targeted vegetable among Malay preschoolers.	Kindergartens (Malaysia)	5-6^2^	The majority of the fathers of the subjects (89.2%) had education up to secondary school, were self-employed (59.5%) and had monthly incomes of below RM1500 (91.9%). Meanwhile, the majority of the mothers were housewives (73%) with secondary education level (86.5%).	The children were exposed to three exposures of round cabbage in the kindergarten setting. The test vegetable had been decided upon based on questionnaire data from the parents. The parents served the vegetable at home once in order to determine the child’s liking of round cabbage.	Not stated
**Ramsey;**[23]	The objective of this study was to compare kindergarteners’ intake of food from a school lunch meal when they are pre-served a larger entrée portion to when they are allowed t0 choose from three preplated entrée portion sizes.	Kindergartens (USA)	2-7_2_	Not stated	A portion size of 4 chicken nuggets was the standard amount offered to the kindergarteners before the study. In the study they were given the choice to self-select smaller entrée portion sizes of 2, 3 and 4 nuggets.	Not stated
**Educational intervention**						
**Baskale et al.;**[24]	The purpose was to develop and implement a program based upon Piaget’s theories. It also determined the average levels of knowledge children would have about nutrition following the program would be different in terms of group, group time, whether there would be any differences in food consumption frequencies between the study and the control group and whether there would be changes in anthropometric measurements of the children.	Nursery schools (Turkey)	5^2^	Different socio-economic layers, but the groups were not significantly different.	Activities were carried out once a week by a nurse educator in the course of 6 weeks. The sessions were carried out in the children’s classroom and the lengths were 20–30 minutes. The themes were the food pyramid, variation of fruits and vegetables, and healthy bones.	Piaget’s cognitive development theory
					Parents in both intervention and control group were given nutrition education in 1 ½ -2 hours.	
**Cason KL;**[25]	The objective of the educational program were to enable preschool children to identify nutritious snack foods, identify and name vegetables, increase willingness to try novel vegetables, help to prepare and consume nutritious foods using developmentally appropriate practices and acquire behaviours that contribute to nutritionally sound food choice and a healthy lifestyle.	Preschool (USA)	4,4^1^	63% African Americans	A theory-based curriculum of 12 lessons of 40 minutes every second week developed for preschool children, the core topics of healthy snacking, fruit and vegetables identification, and the Food Guide Pyramid.	Multiple intelligence theory.
				37% Caucasians		
**Cespedes;**[26]	The objective was to implement and evaluate a nutritional and physical activity educational intervention in preschools.	Preschools (Colombia)	3,7_1_	Low-income: 58%	Children were provided educational and interactive classroom activities throughout 5 months (1 hour daily). Parents participated in 3 workshops and weekly healthy messages were distributed. Teachers participated in 3 centralised workshops and 2 hourly personalised sessions every 14 days. Teachers also received a guidebook.	Social cognitive theory and the trans-theoretical model
				Middle-income: 42%		
**Gorelick et al.;**[27]	To develop a developmentally age appropriate educational curriculum and assess the success of the project curricular objectives.	Preschools (USA)	3-5^2^	Primarily Caucasians	A kit with assessment instruments, fifty classroom activities, patterns to make materials for the classrooms lessons, a recipe book and two film strips. The educational curriculum was delivered by teachers.	Piaget’s developmentally theory
				Wide SES range		
**Hu et al.;**[28]	To evaluate the impact of nutrition education in kindergartens and to promote healthy dietary habits in preschool children.	Kindergarten (China)	4-6^2^	Low-income: 14%	Monthly nutrition education sessions were held over two semesters. The nutrition educational program consisted of a flexible curriculum for children and parents. An illustrated book to all children and pamphlets were delivered to parents. Two series of promotional pictures providing information regarding nutrition were shown to the children.	Not stated
				Middle-income: 57%		
				High income: 29%		
**Johnson SL;**[29]	Objective was to investigate whether children could be taught to focus on internal cues of hunger and satiety and consequently improve their self-regulation of energy intake.	Preschool (USA)	4,7_1_	Primarily high socio economic population.	Introduction of hunger through video and role-play with adults and dolls. Children were instructed before, during and after eating to attend to cues of hunger and satiation.	Not stated
**Nemet;**[30,31]	To examine the effects of a randomized school-based intervention on nutrition and physical activity knowledge and preferences, anthropometric measures and fitness in low socioeconomic children.	Kindergarten (Israel)	3-6^2^	All kindergartens were situated in a low-socio economic area.	Three all-day seminars for teachers. Parents were invited for two health-day festivals. The nutritional intervention was designed mainly to improve nutritional knowledge and was delivered by preschool teachers. Monthly pamphlets with nutrition information were sent home via the children, who were asked to present the nutritional information to their parents.	Not stated
**Parcel et al.;**[32]	To evaluate the impact of a health curriculum on educational and behavioural outcomes	Preschool (USA)	2-4^2^	Not stated	Health education curriculum that was designed to teach selected age-appropriate types of behaviour that enables children to assume greater responsibility for their own health. The classes were taught everyday by a project employee, 1 teacher and 2 aides. The teachers additionally received two-three in-house training.	Social Learning theory
**Piziak V;**[33]	The purpose was to test the effectiveness of a bilingual nutrition game to increase the servings of healthful foods particular vegetables, fruit and water offered to children and decrease the servings of sugar sweetened beverages in the Head Start population.	Preschool (USA)	3-5_2_	The study took place at Head Start, a governmentally funded locally operated school for low-income families. Mexican-American: 57,3%	A pictorial nutrition education game played at class and during meals, the game lends itself to nutrition education. The cards and boards show colour images of culturally appropriate foods and the reverse side gives the name in English and Spanish which may also be used to improve reading skills.	Not stated
**Sirikulchaya-nonta et al.;**[34]	To evaluate the use of food experience, multimedia, and role models for promoting fruit and vegetable consumption.	Kindergarten (Thailand)	4-5^2^	Not stated	The program consisted of 11 activities of 30–40 minutes duration that presented information on health benefits of F&V as manner to improve familiarity with and acceptance of the concept. Teachers, peers, and parents were used as role models while eating together. A take-home letter was sent to the parents once.	Social Learning theory
**Witt et al.;**[35]	Determine whether an interactive nutrition and physical activity program for preschool children increases fruit and vegetable consumption	Child care centres (USA)	4-5^2^	Not stated	The *Color me healthy* program was implemented for 6 weeks; 2 circle-time lessons and 1 imaginary trip were taught to children each week. The lessons were 15–30 minutes in duration.	Not stated
**Multicomponent**						
**Bayer et al.;**[36]	The intervention focused on improving health behaviour on a daily basis in the day care setting, aiming at establishing a health promoting behaviour patterns that might also be maintained outside of the day care setting.	Kindergarten (Germany)	3-6^2^	Children: German nationality:	A behavioural intervention programme using a box-set with activities for kindergarten teachers. Included 2 day training session for KG teachers and a hotline for additional advice. Newsletters for parents was provided and availability of fruit, vegetables and water as well. An internet platform with additional information was established.	Not stated
				Intervention: 91,6		
				Control: 92,4%		
				Parents: Educational level medium – high:		
				Intervention: 73%		
				Control: 71%		
**Brouwer et al.;**[37]	The purpose of this study was to assess the feasibility of a garden-based intervention to promote fruit and vegetable intake among children attending childcare.	Childcare centres (USA)	4,8^1^	Child care directors:	A garden-based intervention with a structured curriculum for child-care providers, consultations by a gardener, and technical assistance from a health educator. The curriculum included an overview module followed by monthly modules designed around a specific crop.	Not stated
				75% African American		
				50% College degree.		
				Children:		
				All centres had children from low-income families		
**De Bock et al.;**[38]	To assess the short-term impact of a nutritional intervention aimed at reducing childhood overweight in German pre-school children.	Kindergarten (Germany)	4,2^1^	Without immigrant background: 65% With immigrant background: 32% Maternal educational level: Low: 16% Middle: 56% High: 21% Missing: 7%	A nutritional intervention, consisting of fifteen 2 hours sessions once weekly over a period of 6 months. Ten modules only targeted children, another five parents and children or parents exclusively. Intervention activities consisted of familiarizing with different food types and preparation methods as well as cooking and eating meals together in groups of children, teachers and parents. Availability of fruit, vegetables and water was increased.	Social Learning theory and Zajonc’ Exposure theory as well as the RE-AIM framework for the process evaluation
**Hammond et al.;**[39]	To evaluate the impact of an early childhood nutrition education program on kindergarten students familiarity with and stated willingness-to-try 16 test foods	Kindergarten (CAN)	5^2^	Cultural inheritance:	Nutrition Educational Program that includes 4 steps; food introduction activities, cooking, journal keeping activity, and communication between child and parents	Not stated
				Intervention: Canadian/British/English: 59% Other: 41%		
				Control: Canadian/British/English: 81% Other: 19%		
**Hoffman et al.;**[40]	The purpose of this study was to examine the impact of a multi-year, multicomponent school-based F&V consumption during school lunch.	Kindergarten (USA)	6^2^	Experimental group: African-American: 29% Latino: 41% Asian: 24% Caucasian: 3% Other: 2% Control:	Multi-year, multi-component fruit and vegetable promotion program, that included school-wide, classroom, lunchroom and family components to promote F & V consumption with an emphasis on F&V in the school lunch. Program components were designed to capture students’ attention and to increase retention of nutrition information using influential role models and deliver consistent messages across the setting.	Social Learning theory
				African-American: 36%		
				Latino: 51%		
				Asian: 0%		
				Caucasian: 4%		
				Other: 9%		
**Vereecken et al.;**[41]	To develop and assist Belgian preschools in the implementation of a healthy school policy and evaluate the impact of the intervention in children’s food consumption.	Preschools (Belgium)	3-4^2^	Intervention: Education low: 60% Education medium: 22% Education high: 18% Control: Education low: 57% Education medium: 26% Education high: 17%	A two-days training was given to school staff. An educational package, including an educational map for the teachers, an educative story and educational material was developed. Food messages and newsletters directed at the school staff and parents were made available.	Intervention Mapping
**Williams et al.;**[42]	To evaluate the effects on a preschool nutrition education and food service intervention	Preschools (USA)	2-5^2^	Minority, primarily African-American: 67% Latino: 33% The majority lived in families with annual income below poverty lines.	There was two intervention types; 1 with food service modification and nutrition education and 1 with food service modification and safety education. The nutrition education segment included a curriculum. The food service modification consisted of help to decrease the consumption of total and saturated fat.	Not stated

^1^Mean; ^2^Range.

### Populations studied

Altogether, 17 of the 26 included studies were North American, three of the studies were carried out in Asia, five in a European context and one study was conducted in South America. Thirteen of the interventions took place in preschools, 10 in kindergartens and three in other facilities where 3 to 6 year-olds were cared for by others than their parents.

### Ethnicity and socio-demographic characteristics of participants

The majority of the single interventions was from the USA and included Caucasians. The educational interventions did not present a clear picture of any tendencies. All of the American multicomponent interventions were targeted towards low-income families or families from African-American or Latino backgrounds. The European interventions targeted children from middleclass families.

### Interventions

Of the single intervention studies identified the majority [10,17-20,22] made modifications to the serving of vegetables, serving either novel or non-preferred vegetables and looked at the effect on vegetable preferences as well as whether peer-models had an influence on the children’s intake during lunch.

We identified eleven interventions consisting of nutritional educational programs carried out either by teachers in the kindergarten, individuals that had undergone a training program or by nutritional educators provided by the research project.

Seven multicomponent interventions included educational activities for the children and delivered similarly to the educational activities described previously. The multicomponent interventions also encompassed other activities like availability of fresh water and fruits and in some cases vegetables [8,36,39] the children participation in growing their own vegetables [22,37], newsletters for parents [36,41], food modifications in the canteen [42] and healthy school policies [41]. A detailed description of the interventions can be found in Table 3.

Table 4 shows the quality assessment and outcomes of interventions.

**Table 4:** Quality assessment and outcome of interventions

**Study**	**Study design**	**Sampling**	**n**	**Duration**	**Limitations**	**Quality1**	**Main target behaviour**	**Primary and secondary outcomes**
Single intervention								
Bannon et al.; [16]	RCT	Convenience	50	3 d	No controlling for internal measurement bias.	**	Children:	The children viewing the gain-framed and loss framed videos were significantly more likely to choose apples than controls. Among the children who saw one of the nutrition message videos, 56% chose apples rather than animal crackers; in the control condition only 33% chose apples.
							Food preference questionnaire.	
							Healthy Food questionnaire (children circled the food they thought were healthy).	
					Short time between exposure and control conditions.			
							Snack choice between an apple or a snack	
					Small sample size			
					Short duration of intervention (3×60 s.)			
Birch LL; [17]	P/P	Convenience	39	4 d	No control	*	Children:	Vegetable preference increased significantly from day 1 to 4.
							Food preferences were assessed	
					No data on allocation short duration of exposure		Food intake of the test vegetables	The total consumption of vegetables decreased during the 4 days, but they still ate the non-preferred food item. Young children were more affected than older children by peer modelling.
					Small sample size.			
O’Connell et al.; [18]	RCT	Randomly	96	6 w		***	Children: Willingness to try new vegetables.	Repeated exposure did not increase vegetable consumption.
								Greater consumption by tablemates was a significant predictor of greater vegetable consumption. 1 gr. of peer intake was associated with roughly 1/5 gr. Intake among the subjects.
Harnack et al.; [19]	Randomized crossover Trial	Not stated	53	6 w	Sampling methodology not stated. Not enough time between exposure/control conditions.	**	Children:	Fruit intake was significantly higher with serving style 1
							Anthropometric measures	
								Vegetables intake did not appear to increase
							Food and nutrient intake during lunch.	
					Small sample size			
Hendy H; [20]	Quasi	Convenience	38	Not stated	Duration not stated	**	Children:	The study found an effect on food acceptance, but the effect had disappeared after 1 month.
							Number of bites taken of the novel foods	
							Food preference	The children serving as peer models rated their food preferences for the novel food higher than the observers.
							Mothers: Information on height, weight, age.	
Leahy et al.; [21]	Quasi	Convenience	77	6 d	No control	**	Children:	<decreasing the energy density of the entrée by 30% significantly decreased children’s energy intake by 25% and total lunch intake by 18%. Children consumed significantly more of the lower-energy-dense version.
							Preference assessment of the two dishes	
							Height and weight.	
							Lunch intake of the two different dishes.	
							Parents:	
							Child feeding questionnaire	
							Socio-demographic variables.	
Noradilah; [22]	Quasi	Convenience, but randomly assigned to intervention	37	3 d	The sample size is small and the duration short. Liking was assessed by parents	**	Children:	The liking scores were significantly higher after the intervention. Consumption of the test vegetable significantly increased from 21.58 to 28.26 on the 3rd day. The effect was especially evident among girls.
							Food intake of the test vegetable	
							Parents: Liking of the test vegetable	
							A questionnaire was developed to obtain information on the usual preparation methods of vegetables, frequency of vegetables served and consumed by children at home	
Ramsey; [23]	Quasi	Not stated	235	5 d	No individual data. Short duration No control conditions Sampling conditions are not stated	*	Children: Food intake at lunch. The food intake was on canteen level, not at an individual level.	Children’s intake of chicken nuggets was greater when they were not given a choice of nugget portion size. Demonstrating that serving larger portion sizes in preschools increase children’s intake of them.
Educational intervention								
Başkale et al.; [24]	RCT	Convenience	115	6 w	High drop-out rate (50% +). No evaluation of parent part of intervention.	***	Children:	Children’s nutritional knowledge increased significantly compared to control group.
							Body Mass Index	
							Mid-upper arm circumference	
							Nutrition knowledge.	
								Healthy food consumption increase significantly in milk, yoghurt, white meat and green leafy vegetables. No anthropometric differences.
							Parents:	
							Demographic data	
							Food consumption of children	
Cason KL; [25]	P/P	Convenience.	6102	24 w	No control or comparison group	**	Children:	Subjects showed significant improvement in food identification and recognition, healthy snack identification, willingness to taste food, and frequency of fruit, vegetables, meat and dairy consumption.
							Knowledge and attitude pictorial questionnaire.	
							Parents: Children’s eating habits and food attitudes.	
							Food frequency questionnaire and pictorial assessment of food likes.	
Cespedes; [26]	Cluster RCT	Randomly	1216	5 m		****	Children: Height and weight Knowledge and attitude scores.	Children showed significantly changes in knowledge and attitudes. Parents showed statistically significant, but minor changes in knowledge, attitudes and habits. More children were eutrophic after the intervention.
							Nutritional status	
							Parents.	
							Parental knowledge and attitudes.	
Gorelick et al.; [27]	RCT	Convenience	187	6 w		***	Children:	The outcomes were fruit identification; vegetable identification; bread identification; vegetables classification; fruit classification; matching; tooth brushing; hand washing; food preparation; food choices and there was a significant improvement over the course of the project. Older children scored higher than the younger ones.
							Identification of bread, fruits and vegetables	
							Food classification of bread, fruits and vegetables.	
							Food preparation	
							Food choices	
Hu et al.; [28]	RCT	Randomly	2102	10 m	Educational intervention not theoretically founded	****	Parents:	No significant difference in anthropometrics but difference in children’s unhealthy diet related behaviours and parents attitudes and knowledge between intervention and control.
							Nutrition-related eating behaviours.	
							Nutrition knowledge	
							Attitudes to the factors they considered important when arranging their children’s dietary habits. Food frequency questionnaire	
							Children: Height and weight.	
Johnson SL; [29]	Quasi	Convenience	25	6 w	Small sample size No control Short exposure time	*	Children: Compensation index based on baseline food intake data. Anthropometric data.	Food intake was measured and showed that children had improved their ability to compensate their energy intake according to the energy density of food offered. The intervention did not have an effect on BMI.
Nemet; [30,31]	RCT	Not stated	725	1 y	Sampling methodology not stated	***	Children: Weight and height. Nutritional knowledge and preferences using a photo-elicitated questionnaire.	Significant increase in nutritional and physical activity knowledge and preference
					Frequency of nutritional education not stated.			
								Significant decrease in number of overweight children.
					Intervention not theoretically founded			
								Significant improvement in fitness
								No sign in BMI percentiles, but 32% from overweight to normal weight.
								At follow-up after 1 year with 206 children BMI and BMI percentiles were significantly lower in the intervention group compared to control. Nutritional knowledge and preferences remained significantly elevated in the intervention group compared to the control.
Parcel et al.; [32]	Quasi	Convenience	173	4 y	Allocation process is missing. Lack of transparency in changes of the sample throughout the study.	**	Mothers: Health values, health behaviour in the home.	No evidence of effect on fruit consumption as a replacement for candy according to parent self-reporting. However, there was strong evidence of less candy eating among the health curriculum group compared to the control according to teacher observation. No evidence of increased variety in food for lunch.
							Children: Health locus of control.	
							Preferences for health and safety behaviour	
					Lack of information on validation			
							Teachers: Observation of children regarding health and safety behaviour.	
Piziak V; [33]	Quasi	Convenience	413	1 y	No control	**	Parents: Food frequency questionnaire.	There was a significant increase in vegetables served outside the preschools both on weeks and in weekends.
					Sampling methodology not stated			
					Intervention not theoretically founded			
					Lack of information regarding intervention group.			
Sirikulchayanonta et al.; [34]	Quasi	Random selected school, but convenient chosen class	26	8 w	Lack of information of intervention group Small sample size.	*	Parents: Demographic variables	The use of food experience, multimedia and role models were effective in increasing F&V consumption
							Family F&V vegetables consumption behaviour.	
							Changes in the children eating behaviour after the intervention.	
							Children: F&V behaviour at lunch time in respect to kinds and amounts consumed.	
Witt et al.; [35]	RCT	Not stated	263	6 w	Sampling methodology not stated.	***	Children: Food consumption of F&V snacks.	Strong evidence that the Color Me Healthy program increased F&V snack consumption among the intervention group compared to the control group. There was a significant increase in consumption of fruit with 20,8% and with vegetable snacks with 33,1%.
					The parental data at follow-up was only at 14%, which was insufficient to make substantive conclusions.		Parents: Changes in children’s F&V consumption at home.	
							Food frequency questionnaire	
							General health survey.	
Multi component	intervention							
Bayer et al.; [36]	Cluster RCT	Randomly	1609	1 y		****	Parents: Children eating habits and food frequency data were examined using a questionnaire.	The program led to an increased proportion of children with high fruit and vegetable consumption after 6 months, which was sustainable with adjusted odds ratios of 1.59 (1.26: 2.01) and 1.48 (1.08:2.03) after 18 months. Subgroup analyses by gender, overweight and parental education, performed in order to assess consistency of effects, showed similar results. Prevalence of overweight and obesity as well as motoric testing results were not statistically different between intervention and control groups.
							Anthropometrics (height, weight) and motoric testing of children were done at the yearly health examination offered to all children in the area of Bavaria.	
Brouwer et al.; [37]	RCT	Randomly	16	4 m	The intervention was carried out in 6 preschool, but only 3 children per class were evaluated causing a small sample size, also the children were not the same at pre and post measurement	**	Children: Structured dietary observation of food intake during meals and snack time in preschools.	Consumption increased with an additional ¼ serving of vegetables, despite fewer vegetables being served.
							Child care centres: Demographic variables including low-income children and ethnicity of child care directors.	
De Bock et al.; [38]	Cluster RCT	Convenience	348	6 m	High dropout rate	***	Children: Height, weight, waist circumference, total body fat using skinfold measurement.	Children’s fruit and vegetable intakes increased significantly.
								No significant changes in the consumption of water and sugared drinks were found.
							Parents: Questionnaire assessing multiple domains of behaviour including	
								No anthropometric measurements changes were found.
							Children’s’ eating behaviour and physical activity.	
							Food frequency questionnaire.	
							Socio-demographic information.	
Hammond et al.; [39]	RCT	Convenience	123	7 m		***	Children: Interviews with children to test their familiarity.	Familiarity with and stated willingness to eat 16 tested foods increased significantly.
							Parents: Demographic variables	
							Children’s willingness-to-eat.	Mentioning of exposure of foods in KG when requesting food at home more than doubled (reported by parents)
							Changes in the child’s dietary habits over the school year.	
Hoffman et al.; [40]	RCT	Convenience	297	1 y	Demographic difference between intervention and control group.	***	Children: Awareness of the intervention	No difference in F&V preferences
							F&V preferences	Increase in fruit and vegetable intake at year 1, but at year 2 a difference was only found on fruit intake.
							Weighed plate waste during 3 lunches in the preschool cafeteria.	
							Height and weight	
							Caregiver/parents: Demographic variables	
Vereecken et al.; [41]	RCT	Convenience	476	6 m	Response rate is low 33%	***	Parents: Food frequency questionnaire on their children’s general food consumption.	Increased fresh fruit intake among the intervention children, but the effect was only significant among parental reported fruit consumption. The increase was due to more available fruit at school.
							Socio-demographic information	
							Questions relating to the school food policy. Teachers: Registration of food available for consumption.	
Williams et al.; [42]	Quasi	Convenience	787	6 m	No information about the allocation process.	***	Children: Dietary intake by observation during school and by interviewing the parents.	Very strong evidence of a decreased relative risk of elevated cholesterol levels among children with elevated cholesterol at baseline in both food service modification groups. Furthermore, strong evidence of a decrease in total cholesterol in the two food service modification groups compared to the control group.
							Weight and height	

*See quality rating scheme in Table 2. Indicating: the the strength of the studies: *Weak, ** Moderate, *** Strong and **** Very strong.

### The study design of included studies

Fourteen of the 26 studies included in this review were randomized controlled studies or cluster randomized controlled trials. Nine quasi-experimental designed studies were found primarily as single or educational intervention [20-23,29,32-34,42]. Only one study used a crossover design as control [19], but neither the sampling method nor the time between intervention and control were stated, making the control effect limited.

### Sampling methods

Random sampling had been used in only five of the 26 studies as most of the studies were based on convenience sampling. Two studies combined random and convenience sampling [22,34]. Four studies did not describe the sampling method used [19,23,30,31,35].

### Sample size

Sample sizes varied greatly between single interventions and the educational and multicomponent interventions. The mean sample size of the single component interventions was 78 and the mean sample size among the educational and multicomponent interventions were 1031 and 522. The mean sample size of all 26 studies was 601.

### Main target behaviours

Food preferences, willingness-to-try novel foods and nutrient intake during lunch were the most used target behaviours in the single interventions. Not surprisingly knowledge and attitudes were the most used target behaviours in the educational interventions, but also consumption of target foods were evaluated using food frequency questionnaires answered by parents. The consumption of target foods were also evaluated in multicomponent studies, but here the intake was measured using observation by researchers or teachers in the setting, just as it was the case for single interventions. Anthropometric measurements of height and weight were applied across the studies, although they only happened in two single interventions [19,20], however it was only used to control for BMI in the statistical analysis. The multicomponent interventions included other anthropometric measures as well.

### Duration of intervention

The single change interventions were relatively short in duration, lasting from 3 to 4 days and up to 6 weeks. The educational interventions with a smaller sample size lasted from 5 to 8 weeks and the studies involving a higher number of participants were of longer duration of between 10 months to 2 years. However, there were exceptions to this, including Cason [25] who evaluated a preschool nutrition program involving 6102 children over 24 weeks and Parcel et al., [32] who carried out a 4 year study targeting approximately 200 preschool children Hendy [20] failed to report their intervention duration. The duration of the multicomponent interventions was generally between 4 and 7 months and up to 1 year.

### Theoretical foundations of interventions

16 of the 26 included interventions did not base their interventions on health behavioural theories. 6 of the studies used Bandura’s social cognitive theory or the related social learning theory. Piaget’s developmental theory was used in 2 studies and others were the theory of multiple intelligences or Zajonc’s exposure theory.

### Information missing from articles

In the single interventions Hendy [20] failed to state the duration of their intervention and Ramsey et al. [23] did not mention their allocation process, however this was due to the study taking place at one canteen without individual data. Nemet et al. [30] and Witt et al. [35] failed to report their sampling process, which was quite surprising considering the high research rigour their studies otherwise presented.

### Bias

The single interventions generally had small sample sizes, lacked controls and were of relatively short duration and with a short period of time in-between the exposure and follow-up measurements and. The majority of studies in both the educational and multicomponent intervention groups suffered from low response rates.

#### Effects of interventions

##### Single intervention

Single exposure interventions failed to demonstrate a significant increase in vegetable consumption. Fruit intake was more easily influenced, however. Results also showed that younger children in particular were influenced by role models and that girls may be more promising role models than boys [17,18].

### Educational intervention

None of the educational interventions resulted in a change in anthropometric measurements, with the exception of [30] who observed a significant decrease in children’s BMI in the overweight children group who became normal weight. At follow-up after one year the BMI and BMI percentiles were significantly lower in the intervention group compared to the control group. Promising results were also found in 6 of the studies where an increase in the consumption of fruit and vegetables was observed. However, none of these changes were significant at the 0.05 level, with the exception of [35], where a significant increase was found in the consumption of fruit by 20.8% and in vegetable snacks by 33.1%. Witt et al. [35] found a significant increase in vegetables served outside preschools, but this was based on mother’s own food frequency data, which may have biased the results [33]. One of the major effects of the educational interventions was in the level of knowledge among its participants. For instance, the level of nutrition-related knowledge increased in two studies [24,30] and the identification of fruits and vegetables increased in two studies [25,27].

### Multicomponent interventions

Six of the multicomponent interventions showed a significant increase in fruit and vegetable consumption, but one found the effect only to be present on fruit consumption after follow-up after 1 year. None of the other studies found an effect on BMI, but one intervention resulted in a decrease in the relative risk of serum cholesterol among children [42]. Only one study [39] evaluated knowledge and found that familiarity with novel foods increased significantly.

## Discussion and conclusions

This review finds that healthy eating interventions can influence the consumption of vegetables through different strategies. The studies acknowledged that a single exposure strategy was insufficient to increase vegetable consumption and that there needs to be an education component as well. This was supported by the fact that the over half of the educational interventions and six of the eight multicomponent interventions resulted in an increase in vegetable consumption. The increase in consumption was greater in the multicomponent studies which could indicate that the more comprehensive the intervention strategy, the more likely the intervention is to be successful.

The effectiveness of the interventions on anthropometric change was more inconclusive, the single interventions did not include measures of BMI and considering how short the duration of their interventions were, it might also be difficult to find change in anthropometric measures. None of the other intervention types that did in fact use anthropometric measurements found an effect on BMI, with the exception of [31]. However Witt et al. [35] found an effect on serum cholesterol.

The educational and the majority of multicomponent interventions included an educational component and the former did find significant increases in nutrition related knowledge, but the multicomponent interventions did not evaluate intermediate effects of knowledge in addition to anthropometrics. This highlights the fact that multicomponent interventions should include measures on knowledge, when they include an educational component, particularly, because the duration of multicomponent interventions often was shorter than the pure educational interventions and anthropometric change is difficult to find during short intervention periods. A lack of follow-up in all of the interventions makes it difficult to conclude whether the observed effects were sustainable over time. With the exception of De Bock et al. [38] and Hoffman et al. [40] the multicomponent and even some of the educational intervention failed either to base or mention the theoretical foundations that they based their educational programmes on. This may be excused in the single interventions that base their studies on empirical data from food choice development theories, but interventions aiming at delivering educational programmes should have some knowledge of health behavioural or educational theories that explains the process behind the success or failure of the implementation of their educational programs. This is again highlighted by the fact that process evaluations were only performed in three of the interventions and the evaluations consisted of either revision of the provided educational materials or checking the adherence to the program, but they did not focus on drivers or barriers behind the implementation of the interventions and thereby to increase the understanding of what made the intervention successful or unsuccessful.

Ethnicity and socio-demographic background play an important role in the development of eating habits and this should be taken into account so interventions are targeted towards those that need it the most. A setting-based approach can be an important intermediate for this, if it is applied to institutions where children of low-income families are nursed and educated. Several educational and multicomponent interventions were targeted towards institutions with children of low-income families and several of them e.g. Cespedes et al. [26], Vereecken et al. [41], and Williams et al. [42] had positive results especially on the consumption of fruits and vegetables that supports the notion of early education establishments as a potential setting to decrease inequalities in health.

### Quality of the evidence

Overall the quality of the intervention studies became better the more comprehensive they were; the single intervention studies were generally of weak quality with small sample sizes, short durations and, in some cases, a lack of controls, which makes it difficult to generalize to a larger population, especially because they were mostly carried out among American Caucasians from families with high socio-economic status. The educational interventions were of better quality and with the largest populations, but still suffered from limitations like lack of consideration in the allocation process, in some cases lack of controls and high drop-out rates. The multicomponent interventions were the most well-designed studies, but also suffered from high drop-out rates and as mentioned above the effectiveness of the educational components were difficult conclude upon, because they failed to evaluate on knowledge. With the exception of Nemet et al. [31] there was a lack of follow-up evaluations that makes it difficult to state whether the outcomes of interventions are sustainable over time.

## Author’s conclusions

### Implications for practice

The majority of interventions found promising results when targeting the consumption of healthy foods or when attempting to increase children’s knowledge of healthy eating, providing sufficient evidence in support of using preschools as a setting for the prevention of chronic disease by making behavioural and lifestyle changes. Interventions are more likely to be successful if they take actions on several levels into account.

### Implications for research

This review supports the need for a longer follow-up of intervention studies in order to assess whether results will be sustainable and how they might influence children’s eating habits later in life. Anthropometric measurements were included in some of the multicomponent interventions but as nutritional status measured as BMI does not change rapidly, interventions using BMI as the outcome measure should be of a longer duration or they should include other intermediate measures such as knowledge and consumption in order to evaluate the effectiveness of the intervention.

Parents may not always be aware of what their children consume outside of the home, or about their knowledge surrounding fruits and vegetables, particularly when children learn about food and healthy eating behaviour in their kindergartens. Even though many choices are made on behalf of the children by their parents at home, children today spend a reasonably large amount of time away from the home environment in day care facilities, together with playmates or cared by other members of family. As a result, a child’s food choice is no longer restricted to being a sole family matter. Children’s knowledge and awareness of food is also being influenced in pedagogical activities, in day care facilities or by talking to their peers. It would therefore be suitable to develop innovative data collection methods, ensuring that the children are able to express what they like to eat and what they know about a given food-related topic. Such innovative research methods should take the developmental stages of the children into account and could perhaps rely more heavily on pictures or on IT material.

The review found that healthy eating interventions in preschools could significantly increase fruit and vegetable consumption and nutrition-related knowledge among pre-school children if the strategy used, is either educational or an educational in combination with supporting component. It further highlights the relative scarcity of properly designed interventions, with clear indicators and verifiable outcomes. Key messages are that preschools are a potentially important setting for influencing children’s food choice at an early age and that there is still room for research in this field. Healthy eating promotion efforts have previously been focusing on schools, but within the last decade the focus have started to shift to pre-schoolers. This review synthesizes some of the interventions that promote healthy eating habits on early education establishments using different strategies. The field of health promotion among this younger age group is still in its earlier stages, but future studies with thorough research designs are currently being undertaken like the Toybox study [10] and The Growing Health Study [43], the healthy caregivers-Healthy children [44] and the Program Si! [45]. These studies may improve our understanding of the effectiveness and underlying mechanisms behind successful implementation of healthy eating efforts in early education establishments.

#### Highlights

• Healthy eating interventions in preschools were classified by their type.

• Comprehensive interventions were more likely to succeed in behaviour change, especially when targeting children of low-income families

• Preschools are a promising venue for increasing fruit and vegetable consumption.

• Evaluations showed a positive increase in food-related knowledge.

• Properly designed interventions, with clear indicators and outcomes are scarce.

## Competing interests

The authors declare that they have no competing interests.

## Authors’ contributions

All authors were involved in the design of the review. MVM performed the literature search. MVM, SH, LRS read and rated the articles. MVM wrote the manuscript with the assistance of SH, LRS and FJPC edited the manuscript. All authors read and approved the final manuscript.
